# Everything’s a Sample: Characterizing Everyday Materials using X-ray Powder Diffraction

**DOI:** 10.1063/4.0000316

**Published:** 2025-04-10

**Authors:** James Kaduk

**Affiliations:** North Central College, Naperville, IL, USA

## Abstract

We can learn something scientifically interesting about literally everything around us by examining it in a powder diffractometer. Comparing a macroscopic understanding of a material with the atomic-scale description proves to be a good way of generating excitement about our science among young people and the general public. I plan to tell stories (case studies) about what can be learned by examining several classes of everyday materials: rocks (including slate and other flooring), water solids, rust and crud (including snow dirt), teeth and fillings, food (sugar, chocolate sandwich cookies, peanut butter), medications (pain relief, decongestant, colonoscopy prep, pharmaceuticals), wood, polymers, and perhaps others.

